# Individual and community-level factors associated with introduction of prelacteal feeding in Ethiopia

**DOI:** 10.1186/s13690-016-0117-0

**Published:** 2016-02-08

**Authors:** Abate Bekele Belachew, Alemayehu Bayray Kahsay, Yemane Gabremariam Abebe

**Affiliations:** School of Public Health, College of Health Sciences, Mekele University, Mekele, Ethiopia

**Keywords:** Prelacteal feeding, Optimal breastfeeding, Multilevel factors, Ethiopia

## Abstract

**Background:**

Ethiopia is a country with low optimal breast feeding practice, and prelacteal feeding is still a norm. Introduction of prelacteal feeding is a known barrier for optimal breast feeding practices. However, knowledge on determinants of introduction of prelacteal feeding is minimal. This study aimed to identify the effects of individual and community-level factors in the introduction of prelacteal feeding in Ethiopia.

**Methods:**

Data for this study was extracted from the nationally representative 2011 Ethiopia Demographic and Health Survey (EDHS) and focused on a sample from child data, with a sample from 576 clusters of 7692 children who were last-born in the past five years preceding the survey. The data was collected using two-stage cluster design, in which enumeration areas forming the first stage and households making the second stage. A two-level mixed effect multivariable logistic regression model was fitted to determine the individual and community-level factors associated with introduction of prelacteal feeding.

**Results:**

From the total sample of children 28.92 % were fed prelacteals. Butter (*n* = 1143), plain water (*n* = 395) and milk-other than breast milk (*n* = 323) were commonly used prelacteals. In multivariable two-level mixed effect model; caesarean mode of delivery (Adjusted odds ratio (AOR) = 1.87; 95 % CI 1.28, 2.73), and late initiation of breastfeeding (AOR = 5.32; 95 % CI 4.65, 6.09) were both positively associated with the odds of giving prelacteals. Higher economic status 28 % (AOR = 0.72; 95 % CI 0.54, 0.98), giving birth at hand of non-health personnel birth assistance (AOR = 0.68; 95 % CI 0.54, 0.87), large birth size of child (AOR = 0.80; 95 % CI 0.68, 0.95) and high community antenatal care use (AOR = 0.58; 95 % CI 0.38, 0.87) were negatively associated with the odds of giving prelacteals. Significant variation in prelacteal feeding practice was also seen among ethnic and religious groups, and across regions.

**Conclusions:**

The prevalence of prelacteal feeding was high that remained a challenge for optimal breastfeeding in Ethiopia. Not only individual-level factors, but also community-level factors contribute to prelacteal feeding practice. Increasing access to health education through increasing maternal health care service coverage and community involvement is crucial.

## Background

Optimal breast feeding (OBF) is an essential nutrition behavior proven to reduce child morbidity and mortality worldwide [1]. It is important for immediate and long-lasting health of child; and has also maternal benefit. Foremost among these is protective of an infant from morbidity by common preventable childhood killers like pneumonia and diarrhea; thereby enhance survival [2]. In the long run, OBF improves child intelligence and protects against long-lasting disease during adult life like diabetes. It also lowers the risk of morbidity and mortality for mothers from time of delivery to their future [1, 2]. Hence, OBF is among most effective interventions that enhance both child and maternal health; thereby reduces health care costs and dependency which in turn promotes economic development of nations [3]. As a result, World Health Organization (WHO), United Nations Children’s Fund (UNICEF) and Ethiopian national public health recommended to put infants to breastfeeding within one hour of birth, exclusively breastfed (EBF) for the first six months of life, and continued breastfeeding up to the age of two years to attain ideal growth, development and health [1, 2]. However, the core indicators of OBF are still low-only 41 % of infants under-six months of age are exclusive breastfed and about 50 % of children initiated breastfeeding early globally [4]. Similarly, only 52 % of children are exclusively breastfed and the same percent of children are initiated breastfeeding early within one hour of birth in Ethiopia [5].

Evidences suggest that prelacteal feeding–providing any food to a newborn before initiation of breastfeeding and/or within three days after birth [2]–is still prevalent cultural practice in many developing countries [6–13], including Ethiopia [5, 14, 15] which is a key contributor to sub-optimal breastfeeding practices [16]. Provision of prelacteal feed to a newborn is a known barrier to continuation of EBF and early initiation of breast feeding which can lead to malnutrition [17–20]. In fact, a child provided with prelacteal feeds is not exclusively breastfed. Prelacteal feeds are often provided for non-nutritional purpose mainly perceived to be useful for smoothing/clearing the throat/bowel [15] in which butter and diluted cow’s milk/sugar are the commonest in Ethiopia [21].

As prelacteal feeds fill a newborn’s small stomach quickly, it interferes with breastfeeding that can in turn reduces breast milk production and enhances early discontinuation of EBF that could finally encourage the provision of prelacteals. Hence, the relationship between breastfeeding and prelacteal feeding is often described as ‘Vicious cycle’ [21, 22]. It also increases the risk of illness like diarrhea and other neonatal infections that may end up with neonatal death [23]. Therefore, understanding factors that are associated with introduction of prelacteal feeds is essential to promote OBF that can reduce both child and maternal morbidity and mortality which in turn improves national mother–child health. However, studies on the determinants of prelacteal feeding are scarce in Ethiopia, and they have limited to specific district in the country with small sample size, thus their findings may not be representative to entire nations [20, 21, 24]. Another weakness of these studies is limitedness to use of analytical techniques that ignores the influence of communities (neighborhoods, cities and regions) on individual decision to provide prelacteal feeds. Therefore, the current study is intended to identify individual and community-level effects on introduction of prelacteal feeding in Ethiopia using a multilevel approach, hence this deeply-rooted norm can be eliminated through a multi-level strategy that incorporates interventions at both individual and community levels. The finding from this study will help the health expertise to articulate a more effective and comprehensive intervention program, whereby intervention is not only targeted at the individual-level, but also at community-level, which in turn permits for possible long term structural change and effective policy making.

## Methods

### Data source and study population

The child dataset of Ethiopia Demographic and Health Survey (EDHS) 2011 a nationally representative cross-sectional survey which was collected using three sets of validated questionnaires was used. The data set was accessed (downloaded) from Measure DHS web site (http://www.measuredhs.com) after registering and stating the purpose of study. The child dataset incorporated all the relevant information on child health from all three sets of questionnaires (a household, a mother’s and a men’s (father’s) questionnaire).

The EDHS 2011 data was based on two-stage stratified cluster sampling. In the first stage of the sampling, the enumeration areas (EA) were selected. An EA is a geographic area consisting of a convenient number of dwelling units which served as counting unit for the census. In stage two, 30 households per EA were selected randomly. A total of 18,720 households were selected. Of these 17,385 eligible women were identified for individual interview, and 16,515 individual women’s surveys completed, making up response rate of 95.0 %. A total of 11,654 children (0–59 months) were included in the survey, and of these 7,692 (weighted) from 576 (weighted) clusters that were last-born five years preceding the survey (in which almost all were less or equal to 3 years of age) and had measurement on their feeding practice within 3-days of birth focused in this study.

To select variables that are appropriate for this analysis the DHS recode-6 manual and questionnaire at the end of EDHS 2011 report were used. Furthermore, details of sampling technique, selection of households, questionnaire, and validation procedure and data quality assurance are published in the survey report [5].

### Study variables

**Fig. 1:**
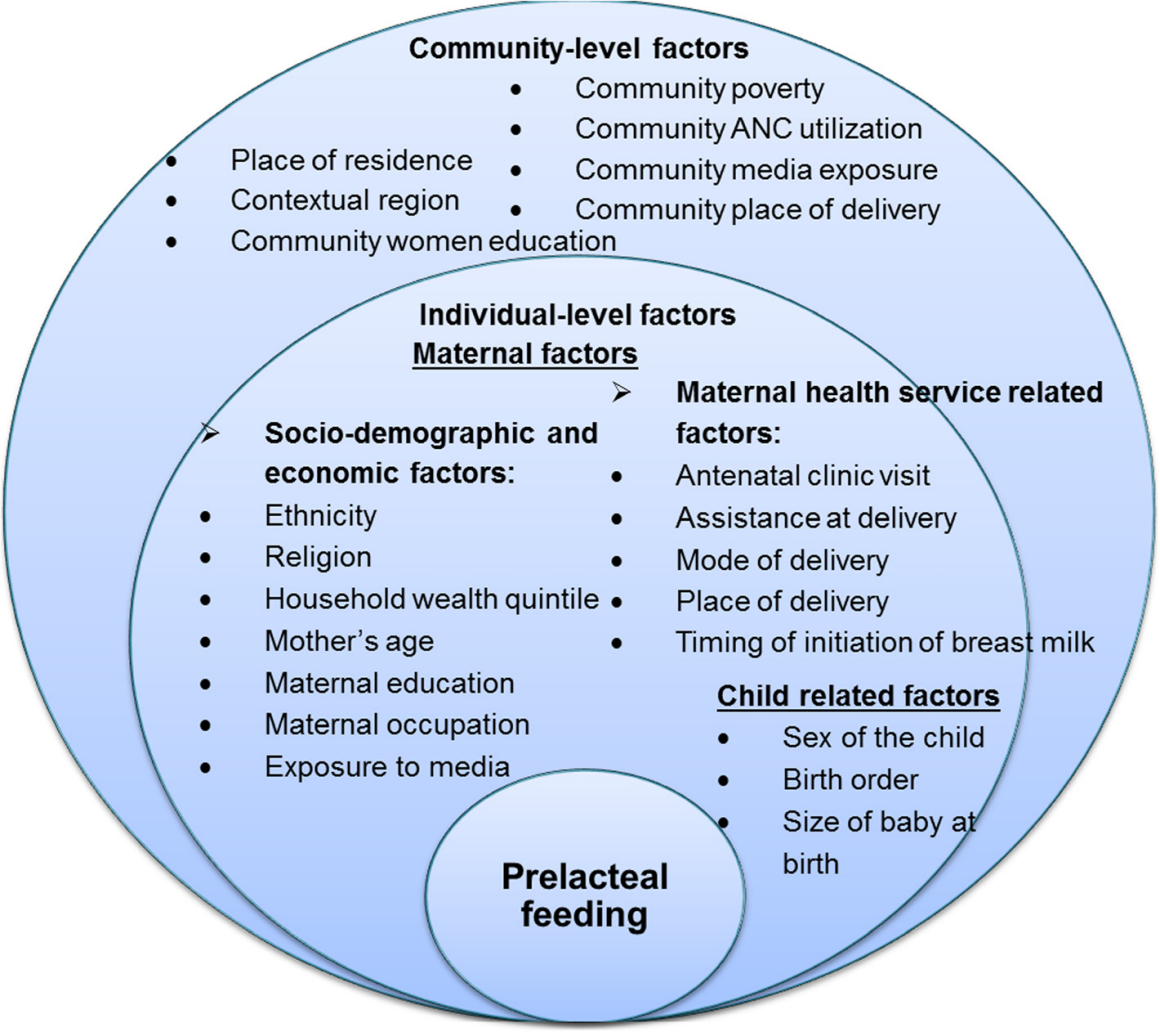
Conceptual frame-work of determinants of introduction of prelacteal feeding

The outcome variable was introduction of prelacteal feeds. The survey counted prelacteal feeds provided within the first three days before mother’s milk for all last-born past five years preceding the survey [5]. The outcome variable coded as 1 = provided prelacteal feeds, and 0 = did not provide prelacteal feeds. Evidence showed that breast feeding practices could be affected by interaction of individual, family, socio-economic, and community factors [25]. In this study, two sets of explanatory variables (individual and community–level) were included. The conceptual framework (see Fig. 1) was used to notice the effect of these factors on the introduction of prelacteal feeding. The characteristics of the community can affect women decision to feed prelacteals directly or modify the relationship between individual characteristics and the decision to fed prelacteals. Similarly, individual-level factors can also modify the effects of community-level factors.

#### Individual–level variables

At this level, both maternal (socio-demographic and maternal health service related characteristics) and child related variables were included.

##### Maternal variables

Ethnicity-Ethiopia is a country with many ethnic groups having different cultural practices living together-was categorized in to five major ethnic groups included in the survey as Afar, Amhara, Oromo, Tigray, Somali and others (Guragie, Berta, Kefficho, Sidama, Wolayita, Hadiya). Given the presence of few respondents on traditional and other categories, religious status was categorized to dominant religious group’s protestant, orthodox, Muslim and others (traditional and others). Household wealth index categorized in quintiles: poorest, poor, average, rich and richest. The index was constructed using household asset data by using a principal components analysis (PCA). Mother’s age at birth was derived by subtracting the date of birth of mother in century month code (CMC) from date of birth of child in CMC. It was then recoded into <25 years, 25–34 years, and > =35 years. Maternal education categorized as no education, primary, secondary, technical/vocational or higher. Given the few respondents in vocational and higher categories, it was categorized in to; no education, primary, and secondary and above. Maternal occupation was assessed by women who are currently working or who have worked in the last 12 months and recorded as not working and other several working categories, and was re-categorized as not working and working. But “not working” doesn’t mean women cannot do any work, rather women limited to household activities.

The survey assessed antenatal care (ANC) visit as the number of antenatal clinic visits. In the current study, it was categorized into no ANC visits, one to three ANC visits, and four or more ANC visits (recommended number of visits). Type of assistance at delivery was categorized as health personnel (i.e. Doctor or Midwife or Nurse) and other person. Mode of delivery was assessed by “Was (NAME) delivered by caesarean?” options were 1 = yes and 0 = no. Place of delivery recoded as hospital (public or private), primary health care centers, health posts, sub health posts, and private clinics. These were categorized as a health facility and the rest (like home, others home) were classified as a home birth. Timing of initiation of breastfeeding (BF) was categorized as less than one-hour (early) and greater than one-hour (late) (based on recommended time to start BF). Exposure to media: EDHS assessed by asking “Do you listen to the radio or watch to television (TV) at least once a week, less than once a week or not at all?”. These variables first categorized to “yes” and “no”, not at all as “no” and else “yes”. Exposure to media variable was considered “yes” if the subject was exposed to one or two of the Medias, and said “no” otherwise.

##### Child related variables

Sex of the child recorded as male and female. Birth order tells about the child’s order of birth, and was recorded as a continuous variable in the EDHS data set. It was categorized as first, second or third, and fourth or more. Size of baby at birth: it was based on the mother’s perception and recorded as very small, smaller than average, average, larger than average and very large. This variable was re-categorized in to: small (very small, smaller than average), average (average) and large (larger than average and very large).

#### Community-level variables

Place of residence and region were non-aggregate community-level variables. Place of residence was recorded as urban and rural. Region was defined as the province where a child is from. Basically, Ethiopia is demarcated for administrative purpose in to 11 regions; these were categorized in to three contextual regions; agrarian, pastoralist and city based on the living status of their population, and settings that may have relationship with infant feeding practice. The regions of Tigray, Amhara, Oromiya, Southern Nations Nationalities & Peoples (SNNP), Gambella and Benshangul Gumuz were recorded as agrarian. The Somali and Afar regions were grouped to form pastoralist region and the city administrations Addis Ababa, Dire Dawa and Harar were grouped to form city.

Another group of community-level variables that were constructed through aggregation from individual-level using an average approaches to conceptualize the neighborhood effect on introduction of prelacteals. Community women education was constructed from education in single year of women and was measured by mean number of years at school among mothers in the specific cluster. Then, it was categorized using national median value to values: low (<=50 % of women below national median year of education) and high (>50 %). The community poverty was measured by constructing a community wealth variable as the proportions of households in the cluster that are in the poorest and poor quintiles of the household wealth index. Then, it was categorized in to; low (<=50 % of women in the poorest and poor quintile of the household wealth index) and high (>50 %). Likewise, Community ANC utilization was created as proportions of mothers with in specific cluster who visited ANC for some number of times. It was then categorized using national level quartiles in to; low ANC utilized community (<=25 % of women are utilizing ANC), middle (25–75 % or intermediate 50 % of women utilizing ANC) and high (> = 75 % of women utilizing ANC). Community media exposure was also created as proportions of mothers who exposed to media with in specific cluster. It was categorized in same fashion as community ANC utilization in to low, middle and high media exposed community. Community place of delivery was measured as proportions of mother who delivered at home with in specific cluster. It was categorized using national median value for the proportion of home delivery in each cluster in to; low (<=50 % of mothers delivered at home) and high (>50 % of women were delivered at home).

### Statistical analysis

#### Multilevel analysis

Study subjects are assumed to be independent in relation to outcome to be studied in the case of traditional regression models. However, when data are structured in hierarchies, units in the same group are rarely independent [26]. The units from same setting (cluster) are more similar among themselves in relation to other units or in terms of the outcome of interest than those from different setting. Hence, it could result in violation of assumption of independence and may have effects of underestimating standard errors and increasing Type I error rates (increases rate of false positivity of our results). In such condition, multilevel modeling can account for factors at individual and community-levels simultaneously and provide a more robust understanding of the factors associated with prelacteal feeding [27]. So that, multilevel models are developed in order to overcome the analytical difficulties that arise when data are organized hierarchically and sampled data is sample of several stages of this hierarchical population, as of EDHS in which children are nested within households, and households are nested within clusters, and there is an intra-group correlation.

Two-level mixed effect logistic regression model was fitted to estimate both independent (fixed) effects of the explanatory variables and community-level random effects on introduction of prelacteal feeding. The first level represents the individual (children) and the second level is the cluster (community). Hence, the log of the probability of introduction of prelacteal feeding was modeled using two-level multilevel model as follows:

$$ \log \left(\frac{\pi_{ij}}{1-{\pi}_{ij}}\right)={\beta}_0 + {\beta}_1{\mathrm{X}}_{ij}+{\beta}_2{\mathrm{Z}}_{ij} + {u}_j $$

Where, i and j are the level 1 (individual) and level 2 (community) units, respectively; X and Z refer to individual and community-level variables, respectively; *π*_*ij*_ is the probability of provision of prelacteal feeds for the i^th^ mother in the j^th^ community; the β’s are the fixed coefficients-therefore, for every one unit increase in X/Z (a set of predictor variables) there is a corresponding effect on the probability mother to provide prelacteal feed. Whereas, *β*_0_ is the intercept-the effect on the probability of mother on provision of prelacteal feed in the absence of influence of predictors; and *u*_*j*_ shows the random effect (effect of the community on mother’s decision to provide prelacteals) for the j^th^ community. By assuming each community has different intercept (*β*_0_ ) and fixed coefficient (β), the clustered data nature and the within and between community variations were taken in to account. To measure the community effect (variation), the intra-community correlation (ICC) is approximated as; $$ ICC=\frac{\sigma_u^2}{\sigma_u^2 + {\sigma}_e^2} $$ where, *σ*_*u*_^2^ denotes community-level variance; and *σ*_*e*_^2^ denotes individual-level variance that is fixed for log distribution to $$ \raisebox{1ex}{${\pi}^2$}\!\left/ \!\raisebox{-1ex}{$3$}\right. $$ (equal to 3.29).

The analysis for this study was conducted using STATA version 12 (STATA Corporation. IC., TX, USA). To adjust for non-proportional allocation of sample to strata and regions, sampling weights were used for estimation of descriptive statistics such as proportions.

As this study used a large data set with several explanatory variables that might be correlated each other, the multicollinearity was checked by using mean variance inflation factors (VIF) and it was 3.29, indicated absence of significant collinearity among explanatory variables. The study also considered multilevel factors which might modify the effect of each other on prelacteal feeding, the interaction effect was checked and no significant interaction effects were seen. The statistical significance was tested using Walid statistics, with results *p*-values less than 0.05 were considered statistically significant. The results of fixed effects (measures of association) were presented as odds ratio (OR) at their 95 % confidence intervals (95 % CIs).

This study approved by the Ethical Review Board of Mekelle University, College of Health Sciences; school of Public Health. The DHS approved by the Ethiopian Health Nutrition and Research Institute (EHNRI) Review Board and the National Research Ethics Review Committee (NRERC) at the Ministry of Science and Technology, Ethiopia.

## Results

### Prelacteal feeding practice

The overall prevalence of prelacteal feeding was 28.92 %. Butter (*n* = 1143), plain water (*n* = 395), milk-other than breast milk (*n* = 323), Sugar or glucose water (*n* = 295) and Sugar-salt-water solution (*n* = 176) were commonly used prelacteals (Table 1).

**Table 1:** Types of prelacteal feeds that were given for children, Ethiopia 2011

Type of prelacteal given	Number^a^
Milk (other than breast milk)	323
Plain water	395
Sugar or glucose water	292
Gripe water	4
Sugar-salt-water solution	176
Fruit juice	2
Infant formula	34
Tea/infusions	65
Honey	8
Fresh butter	1143
Fenugreek	42
Other	116

Note: ^a^Multiple responses were possible and individual-level sampling weights were used

### Background individual and community-level characteristics

Higher proportions of mothers of children were Oromo and Amhara ethnic origin, 34.96 % and 28.57 % respectively. Orthodox was the religion in which majority of participants (42 %) are following, followed by Muslims (32.29 %). The proportion of children was nearly equal across wealth quintiles, from 21.92 % in the poorest to 16.83 % in the richest (Table 2).

**Table 2:** Introduction of prelacteal feeding by background individual and community-level characteristics of children (*N* = 7692), Ethiopia 2011

Variables	Received prelacteal; %	Total; *N* (%)
Individual-level variables		
Ethnicity		
Afar	26.05	62(0.80)
Amhara	44.94	2,198(28.57)
Oromo	24.92	2,689(34.96)
Somali	74.07	184(2.39)
Tigrie	26.38	508(6.61)
Others ethnic groups (Guragie, Berta…)	13.66	2051(26.67)
Religion		
Protestant	10.26	1,727(22.46)
Orthodox	38.14	3,234(42.04)
Muslim	30.51	2,484(32.29)
Others (traditional…)	22.59	247(3.21)
Household wealth quintiles		
Poorest	38.31	1,686(21.92)
Poorer	31.06	1,659(21.57)
Middle	27.27	1,588(20.65)
Richer	22.36	1,465(19.04)
Richest	23.37	1,294(16.83)
Mothers age at birth		
Less than 25 years	30.96	3,000(39.00)
25–34 years	25.39	3,431(44.60)
35+	33.64	1,261(16.39)
Mothers level of education		
No education	31.5	5,141(66.84)
Primary education	23.16	2,198(28.57)
Secondary & higher	27.12	353(4.58)
Mothers has job		
No	26.81	3,486(45.32)
Yes	30.66	4,206(54.68)
Mothers media exposed		
No	32.43	3,130(40.69)
Yes	26.51	4,562(59.31)
ANC visit		
No visit	32.41	4,425(57.53)
1–3 visits	26.31	1,816(23.60)
> = 4 visits	21.52	1,451(18.86)
Delivery assistance		
Other person	29.9	6,739(87.61)
Health personnel (Midwife, Nurse …)	21.97	953(12.39)
Mode of delivery		
Other mode	28.85	7,564(98.33)
Caesarean delivery	32.67	128(1.67)
Place of delivery		
Health facility	21.86	875(11.37)
Home	29.82	6,817(88.63)
Initiation of breastfeeding		
Early	16.16	4,043(52.56)
Late	43.05	3,649(47.44)
Perceived size of child at birth		
Small	34.96	2,405(31.26)
Middle	25.93	3,019(39.25)
Large	26.47	2,268(29.49)
Sex of child		
Female	27.51	3,735(48.56)
Male	30.24	3,956(51.44)
Birth order		
1^st^	33.86	1,357(17.65)
2nd and 3^rd^	27.03	2,401(31.21)
4^th^+	28.36	3,934(51.14)
Community-level variables		
Place of residence		
Urban	25.06	1,132(14.72)
Rural	29.58	6,560(85.28)
Contextual region		
City dwellers	26.66	227(2.95)
Agrarian	27.75	7,202(93.63)
Pastoralist	62.65	263(3.42)
Community ANC utilization		
Low	38.04	2,515(32.70)
Middle	25.83	4,326(56.24)
High	17.65	851(11.06)
Community media exposure		
Low	37.00	1,582(20.57)
Middle	27.00	5,315(69.10)
High	21.00	795(10.33)
Community women average year of schooling		
Low	33.02	4,830(62.79)
High	21.99	2,862(37.21)

Note: Number and Percentages were weighted using individual-level sampling weights

Regarding maternal health service access, about six in ten mothers (57.53 %) were not visited ANC clinic during their pregnancy and the rest visited at least one or more times. Higher proportions of mothers (87.61 %) were delivered by person other than health personnel. The lowest percent of mothers delivered via cesarean section (1.67 %) and the rest were delivered by other modes of delivery. More than half of children (52.56 %) initiated breast feeding early (within one hour of birth). Higher proportions of children (39.25 %) were perceived to be medium in their size during birth (Table 2).

Majority of participants were from agrarian region (93.63 %). More than half of children (56.24 %) were from the community with middle proportions of women using ANC and 69.10 % were from community with middle proportions of women were exposed to one or more media(s) (television and radio). Majority (62.79 %) of women were from community with low average year of schooling (Table 2).

### Multilevel analysis of predictors of introduction of prelacteal feeding

At individual-level; ethnicity, religious status, household wealth status, assistance at delivery, mode of delivery, timing of initiation of breastfeeding and size of child at birth were significantly predicting introduction of prelacteal feeding (Table 3). The odds of introducing prelacteals was 5.46 times (AOR = 5.46; 95 % CI 3.07, 9.72), 7.78 times (AOR = 7.78; 95 % CI 4.56, 13.24) higher among Amhara, and Somali ethnic groups women, respectively than those women from Afar ethnic origin when other variables in the model were controlled. The odds of introduction of prelacteals were significantly high among women of Orthodox (AOR = 1.82; 95 % CI 1.32, 2.48) and Muslim (AOR = 1.48; 95 % CI 1.07, 2.06) religious groups when compared with women from protestant religious groups. The odds of introducing prelacteals also declined as wealth quintile increase from poorest to richest. Accordingly, women from richest wealth quintile had 28 % (AOR = 0.72; 95 % CI 0.54, 0.98) lower odds to introduce prelacteals to their newborn than women from poorest wealth quintile. The odds of introducing prelacteal feeding was 32 % (AOR = 0.68; 95 % CI 0.54, 0.87) lower among women who give birth at hand of health personnel than those deliver at hand of non-health personnel. The odds of introduction of prelacteal feeding was 87 % (AOR = 1.87; 95 % CI 1.28, 2.73) higher among children delivered via cesarean section. Initiating breastfeeding late has significant positive association with giving prelacteal feeding. Those children who initiated late had 5.32 times (AOR = 5.32; 95 % CI 4.65, 6.09) higher odds to receive prelacteal feed than their counterparts. The size of child at birth was negatively associated with the odds of receiving prelacteal feeding. Those children who were medium in size had 17 % (AOR = 0.83; 95 % CI 0.72, 0.97) and those large in size 20 % (AOR = 0.80; 95 % CI 0.68, 0.95) lower odds to receive prelacteal feed than children who were small, respectively.

**Table 3:** Factors associated with introduction of prelacteal feeding in Ethiopia 2011 (*n* = 7540)

Variables	AOR (95 % CI)
Individual-level variables	
Ethnicity	
Afar	1.00
Amhara	5.46(3.07, 9.72)***
Oromo	3.94(2.24, 6.90)***
Somali	7.78(4.56,13.24)***
Tigrie	3.25(1.69, 6.25)***
Other ethnic groups	3.06(1.72, 5.45)***
Religion	
Protestant	1.00
Orthodox	1.82(1.32, 2.48)***
Muslim	1.48 (1.07, 2.06)*
Others (traditional & others)	1.04 (0.64, 1.68)
Household wealth quintiles	
Poorest	1.00
Poorer	0.88(0.72, 1.08)
Middle	0.79(0.64, 0.99)*
Richer	0.77(0.62, 0.97)*
Richest	0.72(0.54, 0.98)*
Delivery assistance	
Other person	1.00
Health personnel	0.68(0.54, 0.87)**
Mode of delivery	
Other mode	1.00
Cesarean delivery	1.87(1.28, 2.73)**
Initiation of breastfeeding	
Early (within 1 h)	1.00
Late	5.32(4.65, 6.09)***
Perceived size of child at birth	
Small	1.00
Middle	0.83(0.72, 0.97)*
Large	0.80(0.68, 0.95)*
Community-level variables	
Contextual region	
City dwellers	1.00
Agrarian	0.37(0.26, 0.53)***
Pastoralist	2.0(1.18, 3.40)***
Community ANC utilization	
Low	1.00
Middle	0.75(0.56, 0.99)*
High	0.58(0.38, 0.87)*

Significant at: *P < 0.05; **P < 0.01; ***P < 0.001. *AOR* Adjusted odds ratio

At community-level, two variables; contextual region and community ANC utilization were significantly predicting introduction of prelacteal feeding. Children from pastoralist region had 2 times (AOR = 2.0; 95 % CI 1.18, 3.40) higher odds to receive prelacteal feeding than children from city region. Children from agrarian region had 63 % (AOR = 0.37; 95 % CI 0.26, 0.53) lower odds to receive prelacteal feeding than city dwelling children. The higher the proportion of women using ANC in the community the likely hood of receiving prelacteals for newborn is low. Children from the community where higher proportions of women use ANC had 42 % (AOR = 0.58; 95 % CI 0.38, 0.87) lower odds to receive prelacteal feeds than those from lower proportions of women use ANC (Table 3).

### Random effects result

The community-level variance (ICC) was 38.96 %, indicating there was a significant difference on introduction of prelacteal feeding at the community-level; the difference declined to 26.72 % when different variables were controlled. Even though the unexplained community-level variance is reduced in combined model, the remaining community-level variance still remains significant; indicating the presence of other factors not addressed in this study (Table 4).

**Table 4:** Community-level variance of two-level mixed effect logit models predicting introduction of prelacteal feeding, Ethiopia 2011

Random effect	Model 1	Model 2	Model 3	Model 4
Community-level variance	2.10*	1.37*	1.81*	1.20*
ICC (%)	38.96	29.39	35.49	26.72
PCV	Reference	34.76	13.80	42.86
Model fit statistics (AIC)^a^	8054	7293	7990	7216

Note: *significant at *p* < 0.001; ^a^AIC (Akaike information criterion); Model 1-Empty (null) model; Model 2- Only individual-level explanatory variables included in the model; Model 3-Only community-level explanatory variables included in the model; Model 4-Combined model PCV (Proportional Change in Variance)

## Discussion

About 29 % of children received prelacteal feeds within the first 3 days of birth which is lower than other previous studies, 41 % in Southern [20], 45.4 % [24] in Eastern and 80 % in North [14] Ethiopia. The possible reason for the inconsistency with southern and northern studies might be due to study participants were only from rural community whereby they might have less access to media and health care, whereas the current study was based on national data. On the other hand, study conducted in eastern Ethiopia was based on sample of mothers-child pairs visiting the public health institutions in specific district of the country and overlooked those children at home. Otherwise, prelacteal feeding is often described as traditional practice related with birth in Ethiopia [21]. The current finding is also lower than reports from other developing countries (35–81.8 %) [6–13]. The main reason could be the difference in context, and health policy our country currently implementing which is mainly focused on prevention with community involvement about different health issues (with especial attention to mothers and infants) through implementing health extension program that works with health development army comprised of the community. Despite the implementation of such program, the current finding suggested the prevalence of prelacteal feeding is still high that could be an implication for low OBF practice in the country, indicating the need to strengthen the program in way to reduce prelacteal feeding practice.

Breastfeeding interventions involving respected members (like religious and community leaders) at each level of breastfeeding promotion programmes are suggested to promote OBF [28, 29]. Likewise, the current study also showed the importance of involving community and religious leaders in breastfeeding promotion programs, in which there was statistically significant difference in the prevalence of prelacteal feeding practice among ethnic and religious groups. This is consistent with other studies conducted in Nepal, Laos and China [8, 10, 30], that reported ethnicity of woman was significantly associated with mothers’ decision to give prelacteal feeds. Similarly, this is consistent with other studies findings in which some religious cultures promote the practice of prelacteal feeding [31–33]. However it is not a religious practice, it is a cultural practice that originated from religious setting [33]. Thus, ethnicity and religions could have their own found on initiation of traditions like practice of prelacteal feeding. Nevertheless, this finding indicates that there is a need to focus on community and religious leaders as information on infant feeding provided by both ethnic and religious group leaders could be more likely to be accepted and changed to practice especially in case of Ethiopia.

The current study also revealed that, the higher household economic status was negatively associated with introduction of prelacteal feeding. This finding is in-consistent with studies conducted in Vietnam and Nepal [22, 34] that reported high socioeconomic status promotes introduction of prelacteal feeding. This could be because of the difference in culture on preferring type of prelacteal feeds. In these studies prelacteals used were costly to be used by lower socioeconomic status; hence only those mothers from high socioeconomic status bought and fed their newborn. However, butter and plain water were more commonly provided prelacteals that can be easily accessible to majority of Ethiopians. Besides, it could be also explained by the fact that higher proportions of women from higher socioeconomic status (SES) are educated and possibly discouraged prelacteal feeding than those from lower SES according to EDHS 2011 report [5].

Giving birth on the hand of health personnel had a negative effect on introduction of prelacteal feeding in the current study, which is consistent with other studies in India and Bolivia [12, 35]. This could be because mothers who delivered on hand of health personnel are more likely to be encouraged and counseled for healthy infant feeding practices. In line with other studies in Egypt, Kuwait and Nigeria [7, 36, 37], the current study also reported cesarean mode of delivery was associated with higher odds of prelacteal feeding. Initiation of breastfeeding within 1 h of birth was associated with lower odds of the introduction of prelacteal feeding. This is consistent with reports of several studies [7, 17, 37]. This might be because those mothers who are late on initiation have miss-perception on colostrum feeding and/or have cultural practice to feed other than breast milk, thus more likely to feed prelacteals. The current study also found that lower size of child was important factor that encourages mothers’ decision to give prelacteal feed, which is consistent with findings of study conducted in Egypt [7]. This could be due to the miss-perception by mothers in which the lower size births could benefit from feeding their newborn with feeds other than breast milk and/or miss-perception that only breast milk can’t meet the nutritional need of the newborn.

The characteristics of community where a woman is living have a significant positive or negative effect on her decision to feed her newborn. The current study found that living in different contextual regions showed significant difference on women decision to introduce prelacteal feed, that is consistent with finding of study in Nepal [34]. This could be explained by difference in living situations, and access to health facilities, media and information across regions. Therefore, this finding indicates that there is a need to focus on reducing differences in access to health care and information while implementing breastfeeding promotion programs.

Antenatal care visit is a best opportunity to promote skilled attendance at birth, and to counsel and educate mothers on essential healthy behaviors like newborn feeding; hence mothers who have visit were more educated or aware of these healthy behaviors and discourage prelacteal feeding [38]. Likewise, as there is higher number of women who visited ANC in a community, the more likely to develop a norm that discourages prelacteal feeding. This effect was indicated by the current study in which living in the community where there is high ANC use discouraged the mother to feed prelacteal to their newborn. Generally, reaching women with health education (counseling) and strengthening the community involvement that currently the government of Ethiopia implementing to increase maternal and child health service coverage can increase the OBF practice and discourage traditional feeding practices like prelacteal feeding.

The current study found that; even if most variation on introduction of prelacteal feeding was explained by individual-level factors, substantial proportion of variation in prelacteal feeding practice was also explained by unmeasured community-level factors. The random effects of the community-level were significant in explaining the prelacteal feeding practice even though it reduced in full model, from 39 % in null model to 26.7 % in full model. This indicates the community-level effect was high and mothers’ decision on giving prelacteal feeding was explained by both individual and community-level factors. However, since the unexplained community variance was still significant after controlling for community variables in the combined model further study should be designed to explore additional community-level factors, and factors evidenced to have effect but not included in this study like knowledge of mother towards breast feeding.

### Strengths and limitations of the study

The current study was conducted by using a multilevel analytical approach that can able to identify the multilevel determinants of introduction of prelacteal feeding and provides important insight to design most appropriate multilevel interventions. Moreover, the results are representative of the entire Ethiopian population because appropriate estimation adjustments such as weighting, accounting for sample design were applied for analysis. Thus, it is the first nationally representative study to report on multilevel factors associated with the introduction of prelacteal feeds. It is also the first to examine the influence of ethnicity and religion on mothers’ decision to give prelacteals to their newborn in Ethiopia.

Despite its strength, the findings of the current study should be interpreted in light of its limitations. Analyses are based on multilevel logit models with random (varying) intercept and fixed coefficients only. Hence, the findings cannot provide evidence of the effects of individual factors variance across communities. The data was collected based on recall that may increase the risk of recall-bias. Finally, EDHS did not collect some information such as maternal beliefs, miss-conceptions and knowledge towards breastfeeding that were evidenced to influence introduction of prelacteal feeding [22, 39], thus their effect was not controlled and seen in this study that might lead to residual confounding.

## Conclusion

The current study showed that the prevalence of prelacteal feeding is high that remained a challenge for optimal breastfeeding in the country. Not only individual-level factors, but also community-level factors contribute to the high prevalence of prelacteal feeding practice. About 39 % of variation in introduction of prelacteal feeding was explained by community-level effects. At individual-level, low socio-economic status, caesarean mode of delivery, giving birth at hand of non-health personnel birth assistance, late initiation of BF and low birth size of child encourage prelacteal feeding practice. In addition, this study also evidenced that there was significant difference in prelacteal feeding practice among ethnic and religious groups. At community-level, low community ANC use encourages introduction of prelacteal feeds, and significant variation in prevalence of prelacteal feeding was also seen across regions. The government should therefore focus in increasing access to health education through increasing maternal health care service coverage and community involvement to increase optimal breastfeeding practices.
